# Prognostic Factors of Chinese Non-small Cell Lung Cancer Patients Receiving Best Supportive Care

**DOI:** 10.7759/cureus.109768

**Published:** 2026-05-27

**Authors:** Joyce May Sum Leung, Ho Ming Cheung, Kwok Sing Ng

**Affiliations:** 1 Clinical Oncology, Prince of Wales Hospital, Hong Kong, HKG; 2 Nuclear Medicine, Queen Elizabeth Hospital, Hong Kong, HKG

**Keywords:** life prognosis, lung tumor, non-small cell lung carcinoma (nsclc), prognosis factors, statistic, supportive and palliative care

## Abstract

Objective:This study aims to identify prognostic factors for Chinese patients with non-small cell lung cancer (NSCLC) receiving best supportive care (BSC) alone.

Materials and methods: This retrospective study investigated patients with newly diagnosed NSCLC who underwent staging fluorodeoxyglucose (FDG) positron emission tomography (PET) and CT at Queen Elizabeth Hospital between 1st January 2018 and 31st March 2024, and were managed exclusively with BSC, without surgery, radiotherapy, or systemic therapy. The inclusion criteria were the following: 1) histologically proven NSCLC, 2) Chinese ethnicity receiving BSC alone, and 3) regular clinical follow-up till death. Subjects were excluded if they had 1) a history of lung neoplasm, 2) a concurrent active neoplasm, 3) defaulted follow-up, or 4) life-threatening events at the first oncology consultation. Potential prognostic determinants were evaluated, including age, gender, performance status, comorbidities, smoking history, previous malignancy, lung tumor histology, serum tumor marker, maximum diameter, as well as maximum standardized uptake value (SUVmax) of the primary tumor, number of metastatic organs, and the presence of pleural/pericardial effusion. Univariate log-rank tests and multivariate Cox proportional hazards regression were performed. The rationale for BSC and the eventual causes of death were analyzed.

Results: Univariate analysis identified male gender, poorer performance status, histology of NSCLC not otherwise specified, larger primary tumor, greater primary tumor SUVmax (higher FDG uptake in primary tumor), and the presence of pleural or pericardial effusion as negative prognostic factors. Subsequent multivariate Cox regression showed four factors retained independent statistical significance: gender, performance status, maximum tumor diameter, and the presence of pleural/pericardial effusion. Three major concerns for the patient's decision to opt for best supportive care were concerns regarding treatment side effects (32.1%), advanced age (25.9%), and poor performance status (17.3%).

Conclusion: This study shows that gender, performance status, tumor diameter, and presence of pleural/pericardial effusion are significant prognostic factors of overall survival in NSCLC patients receiving BSC exclusively.

## Introduction

Non-small cell lung cancer (NSCLC) has the highest incidence and mortality rates of malignancies worldwide [1,2] and in the Hong Kong region [3]. Multiple personalized treatment modalities are available, depending on patient status and tumor characteristics, such as staging, histology, molecular status, PD-L1 expression, etc. [4-6]. In the early stage, radical treatments typically involve surgery and radiotherapy, with or without adjuvant systemic therapy [4]. In advanced stages, treatment options include targeted therapy, immunotherapy, chemotherapy, and radiotherapy [4-6]. While diverse treatment modalities are available, some patients still opt for best supportive care (BSC) alone.

Previous prognostic systems for NSCLC usually focused on early stages [7-9]. However, around 70% of the patients present with stage III to IV disease [10]. Many of the prognostic systems for advanced disease are centered on first-line treatment response [11]. Age, gender, performance status, nutritional parameters, smoking history, comorbidities, tumor, nodes, metastasis (TNM) staging, histopathology, tumor molecular profiles, gene signature, and tumor metabolic activity in fluorodeoxyglucose (FDG) positron emission tomography (PET) and CT were among many potential prognostic factors under investigation [8,10,12-16].

Limited investigations explored the prognostic factors of treatment-naïve patients pursuing BSC alone. While previous prognostic investigations were mostly Caucasian-based, Chinese patients are known to have a younger age at onset, a higher proportion of females and never-smokers, a higher prevalence of EGFR mutations, and longer overall survival (OS) [17-19]. The potential parameters affecting OS have not been well understood. The current study aims to evaluate the prognostic factors determining the OS in Chinese NSCLC patients managed with BSC alone.

## Materials and methods

Patient recruitment

This retrospective study, approved by the Research Ethics Committee (Kowloon Central/Kowloon East) (approval no. KC/KE-19-0048-ER-4), recruited subjects with newly diagnosed NSCLC who underwent staging FDG PET/CT at the Queen Elizabeth Hospital (Hong Kong, CHN) between 1st January 2018 and 31st March 2024. The inclusion criteria were 1) histologically proven NSCLC; 2) Chinese ethnicity with a documented decision for BSC alone with no subsequent surgery, radiotherapy, or systemic therapy (e.g., targeted therapy, immunotherapy, or chemotherapy); and 3) regular follow-up by oncologists/palliative medicine physicians till succumbing. Subjects were excluded if they 1) had a history of lung neoplasms, 2) had a concurrent active neoplasm, 3) were lost to follow-up, or 4) encountered life-threatening events (e.g., myocardial infarction, severe sepsis) at the first oncology consultation. Demographics, including gender, age, Eastern Cooperative Oncology Group (ECOG) performance status assessed at the first consultation, smoking history, number of comorbidities (e.g., diabetes, renal failure), and history of treated non-pulmonary malignancies (e.g., colorectal tumor) were recorded. The characteristics of lung neoplasms were documented, including histology, molecular profile, and PD-L1 expression. Baseline serum carcinoembryonic antigen (CEA) levels within eight weeks of the first visits were documented. The rationale for pursuing BSC alone was analyzed. All patient data were retrieved via the electronic patient record system.

Staging of FDG PET-CT

The FDG PET/CTs were performed within eight weeks of the first oncology consultation. Images were acquired 60 minutes after FDG administration (average activity = 10 mCi) using GE Discovery 710 (GE Healthcare, Illinois, CH, USA) at the Queen Elizabeth Hospital. The maximum 3D length of the primary tumor was measured. The maximum standardized uptake value (SUVmax) was determined by drawing a 3 cm-diameter spherical region of interest over the primary tumor using AW Volume Viewer 4.7 (GE Healthcare). In case of multiple lung lesions, the largest one was regarded as the primary neoplasm. A nuclear medicine physician with over 10 years of experience performed all measurements to ensure consistency. In case of multiple lung lesions, the largest one was regarded as the primary neoplasm. The presence of nodal, intrapulmonary, and distant metastases was documented. The number of metastatic organs was measured (e.g., a patient with nodal, intrapulmonary, and liver metastases was assigned a number of three).

Regular follow-up

After the first consultation, patients had regular follow-up for BSC by oncologists or palliative medicine physicians, typically every one to three months, for symptom management until death. Their dates and eventual causes of death were evaluated. The OS was defined as the time interval from the first consultation to the date of death.

Statistical analysis

Twelve potential prognostic factors were first evaluated in univariate analysis, with variables dichotomized at median or mean: age ($\le$77 vs. >77 years old), gender (male vs. female), ECOG performance status ($\le$1 vs. >1), smoking history (never vs. ever smoker), number of comorbidities ($\le$2 vs. >2), history of treated non-pulmonary malignancy (no vs. yes), histology (adenocarcinoma/squamous cell carcinoma vs. NSCLC not otherwise specified), primary tumor maximum length ($\le$50 vs. >50 mm), primary tumor SUVmax ($\le$13 vs. >13), number of metastatic organs ($\le$1 vs. >1) and the presence of extranodal metastases (yes vs. no), the presence of pleural and/or pericardial effusion (absent vs. present), and serum CEA level ($\le$10 vs. >10 ng/mL).

The OS was evaluated using the Kaplan-Meier method [20], with differences between groups compared using the log-rank test in univariate analysis. Factors having a p-value < 0.1 in univariate analysis were subsequently included in the multivariate Cox proportional hazard regression [21] to identify independent prognostic factors. Statistical significance was defined as p < 0.05. Statistical evaluations were performed using SPSS Statistics version 20 (IBM Corp., Armonk, NY, USA).

## Results

A total of 81 Chinese subjects were included in this study. Their ages ranged from 54 to 93 years old (median 77). Of these, 62 (76.5%) were male, and 19 (23.5%) were female. Most subjects had an ECOG performance status of 0 to 1 (67.9%), while 32.1% had performance statuses of 2 to 3. For smoking history, 76.3% were active/ex-smokers, and 35.8% had never smoked. The number of comorbidities ranged from one to 10, and the median was two. Most of the subjects (85.2%) had no history of malignancy. At the time of analysis, all subjects had succumbed. The OS ranged from 38 to 2072 days, with a median of 485 days (95% confidence interval: 386 to 584 days). Table 1 shows the characteristics of the study population.

**Table 1:** Characteristics of the study population The percentages are calculated based on the total sample size (n = 81). OS: Overall survival

Characteristics		Number (%)
Age (years): 54 to 93 (median 77)	≤77	39 (48.1)
	>77	42 (51.9)
Gender	Male	62 (76.5)
	Female	19 (23.5)
Performance status	0	9 (11.1)
	1	46 (56.8)
	2	19 (23.5)
	3	7 (8.6)
Smoking history	Never	29 (35.8)
	Active smoker	20 (24.7)
	Ex-smoker	32 (39.5)
Number of comorbidities		1 to 10, median 2
History of cancers	No	69 (85.2)
	Yes	12 (14.8)
OS		38 to 2072 days, mean 485

Table 2 shows the tumor characteristics. The most common histology was adenocarcinoma (53.1%), followed by squamous cell carcinoma (27.2%) and NSCLC not otherwise specified (16.0%). For EGFR mutation status, 56 out of 81 (69.1%) subjects were wild type, 18 (22.2%) were unknown, and seven (8.7%) had mutations (three had G719X, two had L858R, one had exon 19 deletion, and another one had exon 20 insertion). For anaplastic lymphoma kinase (ALK) status, no ALK rearrangement was detected: 60 (74.1%) subjects were wild type, and the remaining 21 (29.5%) were unknown. For PD-L1 expression, 17 (21.0%) were negative (i.e., tumor proportion score <1%), 10 (12.3%) were positive, and 54 (66.7%) were unknown. Among the 10 subjects with positive PD-L1, 2 (20%) had PD-L1 > 50%. No subgroup OS analysis was performed for EGFR, ALK, or PD-L1 because of the limited number of subjects with mutations.

**Table 2:** Tumor characteristics of patients with NSCLC The percentages are calculated based on the total sample size (n = 81). NSCLC: Non-small cell lung cancer, SUVmax: Maximum standardized uptake value, CEA: Carcinoembryonic antigen

Tumor characteristics		Number (%)
Histology	Adenocarinoma	43 (53.1)
	Squamous cell carcinoma	22 (27.2)
	Adenosquamous	1 (1.2)
	Poorly differentiated	2 (2.5)
	NSCLC not otherwise specified	13 (16.0)
Maximum tumor length (mm)		13 to 119, mean 52.9
Primary Tumor SUVmax		1.3 to 34.1, mean 13.3
Number of metastatic organs		0 to 6 (median 1)
Lymph node alone		25 (30.9)
Extranodal metastases		49 (60.5)
Effusion	No	61 (75.3)
	Pleural	17 (21.0)
	Pericardial	1 (1.2)
	Both	2 (2.5)
Serum CEA (ng/mL)		1 to 1464, median 10

Based on the staging PET/CT, the maximum tumor length ranged from 13 to 119 mm, with a mean of 52.9 mm. The primary tumor SUVmax ranged from 1.3 to 34.1, with a mean of 13.3. The number of metastatic organs varied from zero to six, and the median was one. The majority had extranodal metastases (60.5%), with no pleural or pericardial effusion (75.3%). Some subjects had early stages without distant metastasis (39.5%). The FDG is well-known to be insensitive for detecting brain or leptomeningeal metastasis because of the intense physiological brain activity [22]. Dedicated brain imaging (contrast CT/MRI) is therefore recommended for thorough staging [23]. In this study, only 22 subjects (27.2%) had dedicated brain imaging, and 11 (50%) of them had brain metastases. As few subjects in this study had dedicated brain imaging, no subgroup analysis for brain metastasis was performed. Serum CEA ranged from 1 ng/mL to 1464 ng/mL, and the median was 10 ng/mL (standard reference range is <5 ng/mL for non-smokers and <10 ng/mL for smokers).

**Figure 1:**
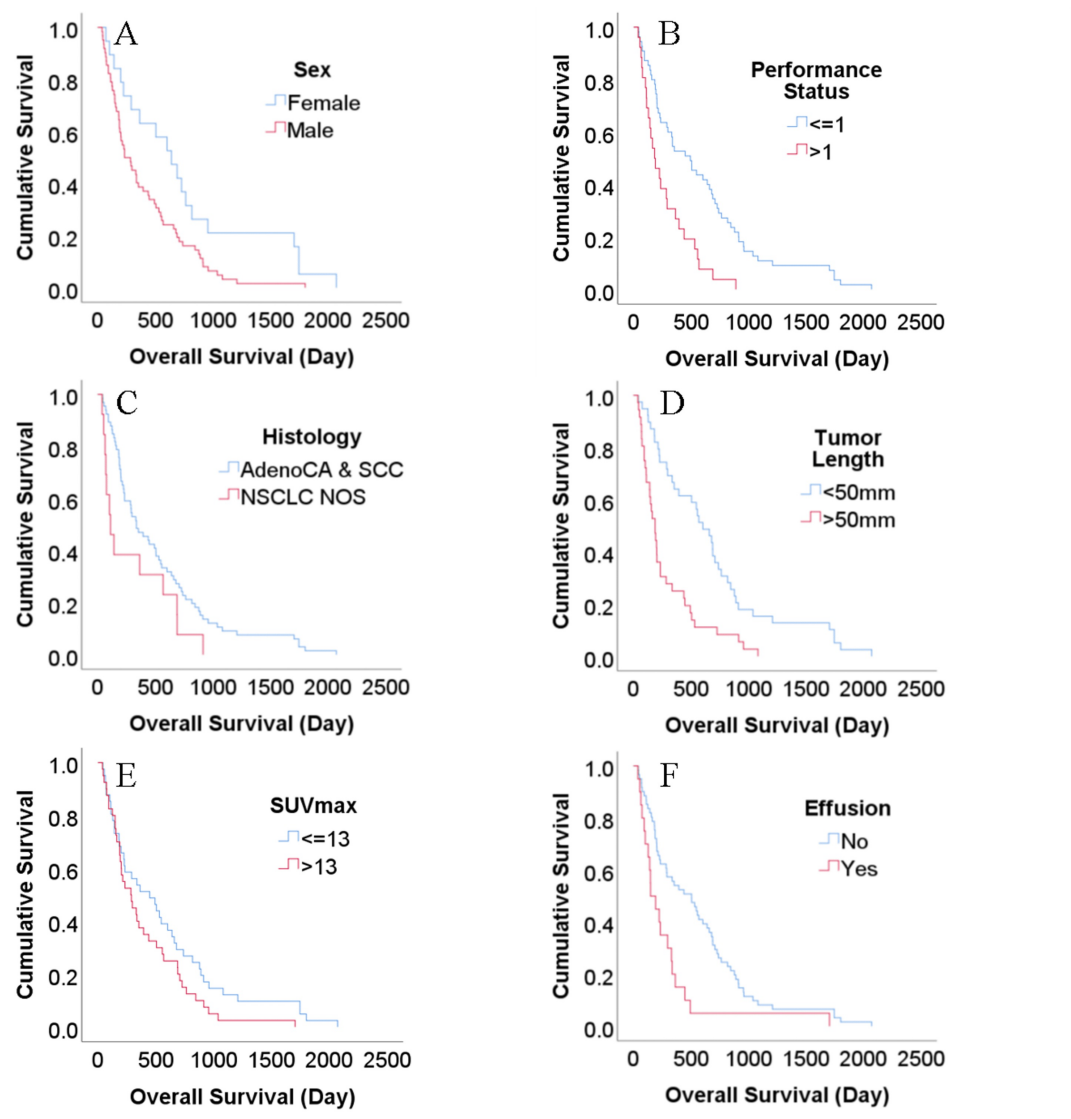
Kaplan-Meier OS analyses Shown are the OS plots for gender (A), performance status (B), histology (C), maximum tumor length (D), primary tumor SUVmax (E), and the presence of pleural and/or pericardial effusion (F). AdenoCA: Adenocarcinoma, SCC: Squamous cell carcinoma, NOS: Not otherwise specified, SUVmax: Maximum standardized uptake value, OS: Overall survival

The results of the univariate log-rank analysis are summarized in Table 3. Of the 12 factors under investigation, six showed potential association with OS (p < 0.1): gender, performance status, histology, maximum length, and SUVmax of the primary tumor, and the presence of pleural and/or pericardial effusion. Significantly shorter OS was noted in patients who were male, had poorer performance status, were diagnosed with NSCLC not otherwise specified, and had larger primary tumors and greater SUVmax and effusion. Figure 1 shows the Kaplan-Meier curves for these six factors. On the other hand, the remaining six factors showed no potential association with OS (p > 0.1): age, smoking history, history of cancers, number of metastatic organs, presence of extranodal metastases, and serum CEA level.

**Table 3:** Univariate log-rank analyses of prognostic factors associated with OS *For histology, the OS comparison was adenocarcinoma and squamous cell carcinoma vs. NSCLC not otherwise specified. The percentages are calculated based on the total sample size (n = 81). NSCLC: Non-small cell lung cancer, SUVmax: Maximum standardized uptake value, CEA: Carcinoembryonic antigen, OS: Overall survival

Variable		OS (days)	p-value
Age (years)	≤77	486	0.822
	>77	484	
Gender	Male	402	0.015
	Female	756	
Performance status	≤1	586	<0.001
	>1	271	
Smoking history	Never	612	0.119
	Active or ex-smoker	431	
Number of comorbidities	≤2	436	0.297
	>2	552	
History of cancers	No	463	0.255
	Yes	609	
Histology	Adenocarcinoma	542	
	Squamous cell carcinoma	474	
	NSCLC not otherwise specified	299	p = 0.049*
Maximum tumor length (mm)	≤50mm	682	<0.001
	>50mm	279	
Primary tumor SUVmax	≤13	567	0.09
	>13	401	
Number of metastatic organs	≤1	544	0.22
	>1	430	
Presence of extranodal metastases	No	514	0.687
	Yes	467	
Effusion	No	552	0.002
	Yes	281	
Serum CEA (ng/mL)	≤10	530	0.229
	>10	420	

Based on the results of the univariate analysis, the potential six factors in Figure 1 were included in the subsequent multivariate Cox proportional hazard regression analysis. Table 4 demonstrates that four variables retained independent prognostic significance (p < 0.05): gender, performance status, maximum length, and effusion. Among the four factors, performance status > 1 was the greatest predictor of mortality (hazard ratio (HR) 2.57), followed by the presence of effusion (HR 2.27), tumor length >50mm (HR 2.25), and male gender (1.91). On the other hand, histology and primary tumor SUVmax did not achieve statistical significance in multivariate analysis (p > 0.05).

**Table 4:** Multivariate Cox regression analyses of prognostic factors associated with OS The HR with 95% CI were included. HR: Hazard ratio, CI: Confidence interval, NSCLC: Non-small cell lung cancer, SUVmax: Maximum standardized uptake value, OS: Overall survival

Variable		HR (95% CI)	p-value
Gender	Male	1.91 (1-3.68)	0.05
	Female	Reference	
Performance status	≤1	Reference	0.001
	>1	2.57 (1.47-4.48)	
Histology	Adenocarcinoma and squamous cell carcinoma	Reference	0.399
	NSCLC not otherwise specified	1.34 (0.68-2.65)	
Maximum tumor length (mm)	≤50mm	Reference	0.003
	>50mm	2.25 (1.31-3.86)	
Primary tumor SUVmax	≤13	Reference	0.539
	>13	1.17 (0.71-1.93)	
Effusion	No	Reference	0.011
	Yes	2.27 (1.20-4.27)	

With the advancement of NSCLC management, multiple treatment modalities are available. Yet, the subjects in this study still pursued BSC exclusively, and their rationales for this decision are listed in Table 5. The top three reasons were concern of treatment side effects (32.1%), advanced age (25.9%), and poor performance status (17.3%). In particular, seven subjects (8.6%) had EGFR mutations. While TKI could have been considered for the seven (8.6%) patients with EGFR mutations, they decided on BSC after detailed discussion with clinical oncologists about TKI indications and possible side effects. Four of them worried about treatment side effects, and the other three were of advanced age (83, 87, and 89 years old). Lastly, 73 (90%) out of the 81 subjects had causes of death identified. The top three causes were pneumonia (76.7%), acute exacerbation of chronic obstructive pulmonary disease (2.7%), and urinary tract infection (2.7%).

**Table 5:** Reasons for pursuing BSC alone The total is greater than 100%, as some subjects had more than one reason for opting for BSC. BSC: Best supportive care

Reasons	Number	Percentage
Treatment side effects	26	32.1
Advanced age	21	25.9
Low performance status	14	17.3
Treatment cost	7	8.6
Asymptomatic	2	2.5
Unknown	14	7.3

## Discussion

Overall survival is one of the major concerns for cancer patients opting for BSC alone. This study shows that performance status, gender, maximum tumor length, and the presence of effusion are independent prognostic factors for OS in NSCLC managed with BSC. Performance status has the highest HR of 2.57 among the four factors. This is one of the expectations because performance status is a standardized clinical assessment for patients' general well-being and ability to perform daily activities. Lower performance status implies poorer physical ability for self-care and is typically associated with shorter life expectancy [12]. For gender, our results align with previous studies showing males have shorter OS across different stages and histology [8,24]. Possible contributing factors have been proposed to explain the gender effect [25], including different smoking histories, health-seeking behaviors, and tumor histology, as well as genetic mutations among males and females. The maximum tumor length contributes to the tumor staging of the International Association for the Study of Lung Cancer (IASLC) TNM classification. The TNM staging system is a standardized method for risk stratification and survival prognosis. The current cutoff of 50 mm incidentally aligns with the T2/T3 threshold in TNM 9th edition [26]. Similarly, the presence of pleural or pericardial effusion is related to M1a (single metastatic site) staging. Malignant effusion is expected to progress in patients pursuing BSC and can potentially impair baseline respiratory function. Thus, these patients may have increasing susceptibility to life-threatening respiratory failure during pneumonia, the predominant (76.7%) cause of death in this study.

The first-line treatments for patients with positive EGFR and PD-L1 are targeted therapy and immunotherapy, respectively. This can explain the very low prevalence of the subjects with positive EGFR (8.6%) and PD-L1 (12.3%) pursuing BSC in this study. On the other hand, the top rationale for pursuing BSC was concern about treatment side effects (32.1%). It remains unclear if fewer patients would opt for BSC in the future as treatment toxicities reduce. 

One major pitfall of this study is the retrospective design, which resulted in non-standardized history-taking and investigations. For example, only 27.2% of subjects underwent dedicated brain imaging for complete staging, potentially underestimating the metastatic burden. Another pitfall is that the limited sample size impeded subgroup analysis, including genetic mutation or PD-L1 status. Furthermore, this study focused on the Chinese population and may not apply to other ethnicities. The effects of confounding factors require further evaluation, including NSCLC staging and arbitrary variable dichotomization.

## Conclusions

This study demonstrates that gender, performance status, length of the primary tumor, and the presence of effusion are independent prognostic factors of OS in Chinese patients with NSCLC managed with BSC exclusively. Patients have shorter OS if they have a performance status >1, pleural and/or pericardial effusion, a primary tumor length >50 mm, and are of the male gender. Among the four factors, performance status appears to be the most important. Closer clinical follow-up can be considered for patients with poorer prognoses to provide better supportive care. 
